# Testing Projected Climate Change Conditions on the *Endoconidiophora polonica* / *Norway spruce* Pathosystem Shows Fungal Strain Specific Effects

**DOI:** 10.3389/fpls.2017.00883

**Published:** 2017-05-26

**Authors:** Riikka Linnakoski, Kristian M. Forbes, Michael J. Wingfield, Pertti Pulkkinen, Fred O. Asiegbu

**Affiliations:** ^1^Department of Forest Sciences, University of HelsinkiHelsinki, Finland; ^2^Department of Microbiology and Plant Pathology, Forestry and Agricultural Biotechnology Institute, University of PretoriaPretoria, South Africa; ^3^Department of Virology, University of HelsinkiHelsinki, Finland; ^4^Natural Resources Institute FinlandLäyliäinen, Finland

**Keywords:** blue stain fungi, carbon dioxide, climate change, *Endoconidiophora polonica*, forest pathogen, *in vivo*, pathogenicity, *Picea abies*

## Abstract

Climate changes, exemplified by increased temperatures and CO_2_ concentration, pose a global threat to forest health. Of particular concern are pests and pathogens, with a warming climate altering their distributions and evolutionary capacity, while impairing the ability of some plants to respond to infections. Progress in understanding and mitigating such effects is currently hindered by a lack of empirical research. Norway spruce (*Picea abies*) is one of the most economically important tree species in northern Europe, and is considered highly vulnerable to changes in climate. It is commonly infected by the fungus *Endoconidiophora polonica*, and we hypothesized that damage caused to trees will increase under future climate change predictions. To test this hypothesis an *in vivo* greenhouse experiment was conducted to evaluate the effects of a changed growing environment on *E. polonica* infected Norway spruce seedlings, comparing ambient conditions to predicted temperatures and CO_2_ levels in Finland for the years 2030 and 2100. In total, 450 seedlings were randomized amongst the three treatments, with 25 seedlings from each allocated to inoculation with one of five different fungal strains or mock-inoculation. Seedlings were monitored throughout the thermal growing season for mortality, and lesion length and depth indices were measured at the experiment conclusion. Disease severity (mortality and lesions) was consistently greater in fungal-inoculated than mock-inoculated seedlings. However, substantial differences were observed among fungal strains in response to climate scenarios. For example, although overall seedling mortality was highest under the most distant (and severe) climate change expectations, of the two fungal strains with the highest mortality counts (referred to as F4 and F5), one produced greater mortality under the 2030 and 2100 scenarios than ambient conditions, whereas climate scenario had no effect on the other. This study contributes to a limited body of empirical research on the effects of projected climate changes on forestry pathosystems, and is the first to investigate interactions between Norway spruce and *E. polonica*. The results indicate the potential for future climate changes to alter the impact of forest pathogens with implications for productivity, while highlighting the need for a strain-specific level of understanding of the disease agents.

## Introduction

Human-related disturbances including climate change and globalization pose novel threats to global forest health (Gauthier et al., 2015; Trumbore et al., 2015; Wingfield et al., 2015; Ghelardini et al., 2016). Although forests can adapt to tolerate and recover from some level of disturbance, adaptation processes are currently undermined by the rate of climate change (Julkunen-Tiitto et al., 2015; Trumbore et al., 2015). Pathogens and pests represent some of the most important threats to forest health where climate change is occurring (Ayres and Lombardero, 2000; Allen et al., 2010; Wingfield et al., 2015; Ghelardini et al., 2016). These factors are recognized as an increasing concern worldwide, but an understanding of how systems will respond to climate change, and the associated need to enact mitigation strategies, is hindered by a lack of empirical research.

Warmer temperatures, elevated CO_2_ levels and longer growing seasons favor many pathogens through enhanced growth rate, reproduction, survival and accelerated evolution (Chakraborty and Datta, 2003; Roy et al., 2004; Chakraborty, 2013). Climate change can also influence plant resistance and tolerance to pests and pathogens (Jamiesen et al., 2012). For instance, research addressing the effects of drought stress on forest ecosystems has indicated the potential for exacerbated epidemics under climate change (Allen et al., 2010; Jactel et al., 2012). Damage caused by pests and pathogens is generally expected to increase under future climate change scenarios (Coakley et al., 1999; Ayres and Lombardero, 2000; Allen et al., 2010; Björkman and Niemelä, 2015), and is supported by model-based simulations (Brasier, 1996; Jönsson et al., 2009; Marini et al., 2012) and some experimental research (Karnosky et al., 2002; Percy et al., 2002; Chakraborty and Datta, 2003; Mitchell et al., 2003; Roy et al., 2004; Váry et al., 2015). Other studies have identified reduced or no effect of climate change on plant diseases (Chakraborty et al., 1998; Coakley et al., 1999; Roy et al., 2004). These effects vary geographically and are often species and strain specific (Coakley et al., 1999; Sallé et al., 2005; Garret et al., 2006), highlighting the need for systems-based research.

Boreal forests in Northern Europe are particularly vulnerable, where predictions for future climate changes are amongst the most severe (Jylhä et al., 2004; Field et al., 2014). Overall, elevated temperatures and atmospheric CO_2_ concentrations are expected to favor tree growth and increase forestry productivity (Bergh et al., 2003; Kellomäki et al., 2008). However, some tree species are sensitive to warming temperatures and drought stress, which could lead to major shifts in tree species distributions (Bonan, 2008; Hanewinkel et al., 2011; Lévesque et al., 2013; Field et al., 2014). An iconic example is the Norway spruce (*Picea abies*), which is one of the most economically important forestry tree species in Europe. This species is considered particularly vulnerable to climate change, as it appears to have a limited capacity to adjust to warming-induced drought conditions (Kellomäki et al., 2008; Lévesque et al., 2013) and increased CO_2_ levels (Sallas et al., 2003; Kroner and Way, 2016). Predicted climate changes are likely to decrease its southern range abundance, with consequent negative impacts on economic potential in some regions (Kellomäki et al., 2008; Lévesque et al., 2013).

One of the most important pests of Norway spruce in Europe is the Eurasian spruce bark beetle (*Ips typographus*). Like many insect species, the bark beetle is highly responsive to changes in temperature (Björkman and Niemelä, 2015), and a warming climate has already had observable effects on its development and voltinism (Jönsson et al., 2009; Økland et al., 2015). The beetle has a symbiotic association with several fungi for which it serves as a vector (Wingfield et al., 1993; Seifert et al., 2013). The most pathogenic fungus associated with the Eurasian spruce bark beetle is *Endoconidiophora polonica*, which is capable of killing mature Norway spruce trees (Christiansen and Horntvedt, 1983; Solheim, 1988; Krokene and Solheim, 1998; Kirisits and Offenthaler, 2002). It is not known how projected climate changes will influence the effects of *E. polonica* on tree health or its ability to facilitate colonization by the bark beetle.

The aim of this study was to experimentally test *in vivo* the effects of a changed growing environment on *E. polonica* infected Norway spruce seedlings, comparing current ambient conditions (for the year 2015) to projected temperature and CO_2_ levels for the years 2030 and 2100 in Finland (Jylhä et al., 2004, 2009). Previous research has demonstrated that seedlings provide an effective model for larger tree health within this system (Krokene and Solheim, 1998; Repe et al., 2015). Due to the general inability of this tree species to adjust to environmental changes, we hypothesized that disease severity on seedlings would be greater under predicted future climate change scenarios than current ambient conditions.

## Materials and Methods

### Plant Material

A total of 450 (2-year-old) Norway spruce seedlings (Fin Forelia Oy, Nurmijärvi) originating from Imatra, Southern Finland (61° 09′ N, 28° 048′ E) were used. Average seedling height at the beginning of the experiment was 40 cm. Seedlings were potted into plastic trays of 36 cells (Plantek 36 F), each 64 mm × 64 mm × 90 mm (depth), filled with fertilized peat (Vapo peat for forest trees). Plant density in the trays was 240 plants/m^2^. All seedlings were maintained in a greenhouse (mean temperature 14.8°C) for 1 week and then transferred to treatment conditions (see below), where they were acclimatized for ∼4 weeks prior to fungal inoculations.

### Experiment Design

The experiment was conducted during the 2015 thermal growing season (between 9 June and 28 October) at the Haapastensyrjä field station of the Natural Resources Institute Finland (Luke) in southern Finland. This area is characterized by a semi-continental climate. During the experimental period, the mean daily temperature ranged from 4 to 15°C, consistent with long-term averages. The exception occurred in August – September, which was approximately 1.5°C warmer than the long-term mean (Finnish Meteorological Institute, 2016). The average ambient CO_2_ level was 402 ppm (at the SMEAR II station (61°51′N, 24°17′E, 4.2 m a.s.l.) (Junninen et al., 2009). The field station is equipped with a commercial climate control system LCC-1200 linked to LCC-1240 using SuperLink v.3.5 software (DGT-Volmatic, Odense, Denmark) to monitor and/or control, e.g., the temperatures and CO_2_ levels outside and inside the greenhouse compartments at intervals of 1 min.

For the experiment, the 450 spruce seedlings were block randomized (in trays) amongst three treatments: (1) outside temperature +1°C + elevated CO_2_, (2) outside temperature +4°C + elevated CO_2_, and (3) outside/ambient temperature and CO_2_ conditions. One hundred and fifty seedlings were allocated to each treatment. These temperatures are based on climate predictions for southern Finland for the years 2030 (+1°C) and 2100 (+4°C) (Jylhä et al., 2004, 2009). CO_2_ levels were set at 550 ppm, consistent with low range predictions.

The two temperature-controlled seedling groups were maintained in separate compartments of a large greenhouse, where the DGT-Volmatic system monitored outside temperatures and increased the greenhouse room temperatures accordingly. The control seedlings were maintained alongside the greenhouse, and were thus subject to very similar conditions to those in the climate scenario treatment groups (including natural day length, with temperature and CO_2_ levels the notable exceptions). However, we cannot exclude the possibility that other potential differences, such as light intensity and quality (Ballaré, 2014), influenced seedling resistance to the fungal infections. Each seedling was inoculated with one of five *E. polonica* strains (Supplementary Table S1), or mock-inoculated (25 seedlings per fungal strain/treatment). The inoculation experiment started at the beginning of July and was concluded 12 weeks later, in October 2015, to encompass the thermal growing season in the region. During the experiment, seedlings were watered as required to maintain moist soil (usually three times a week). No additional fertilization was given. The trays were rotated every 3 weeks to minimize potential effects due to their positioning.

### Fungal Inoculations

*Endoconidiophora polonica* strains were isolated from adult European spruce bark beetles (*I. typographus*) and their phoretic mites collected from outbreak regions of Finland and neighboring Russia (Linnakoski, 2011; Linnakoski et al., 2016). For the purposes of this study, fungal strains are designated F1 – F5, and further described in Supplementary Table S1. Strains were plated on 2% Malt Extract Agar (MEA; 20 g Bacto^TM^ Malt extract and 20 g Difco Bacto^TM^ agar from Becton, Dickinson and Company, Sparks, MD, United States, and 1000 ml of MQ-water) in 9 cm Petri dishes. Cultures were grown in an incubator at 25°C for 1 week prior to being used for the inoculations.

**FIGURE 1:**
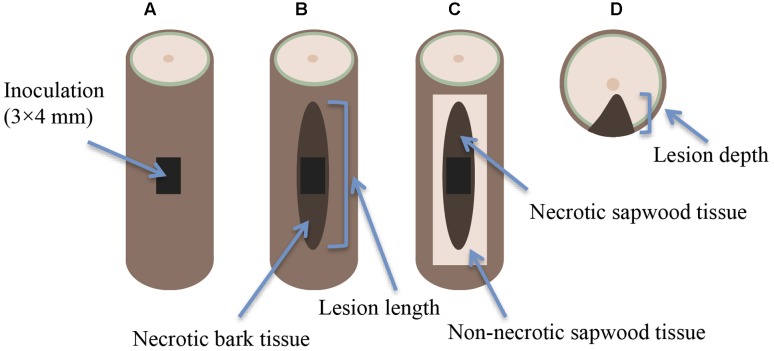
**Lesion indices on the seedling stem in relation to the inoculation:** **(A)** inoculation point **(B)** necrotic lesion on the outer bark (bark lesion), **(C)** necrotic lesion along the sapwood surface (sapwood lesion), and **(D)** lesion depth.

Seedling inoculations were made approximately half way up the stems (on the 1st-year shoot) by cutting a bark flap (3 × 4 mm) with a sterile scalpel (**Figure 1A**). An inoculum of approximately the same size was cut from the actively growing outer zone of the culture plate, and placed onto the wound with the mycelial surface facing the exposed sapwood region. The inoculum site was then covered with the bark flap and sealed with Parafilm to prevent contamination and desiccation. The Parafilm was removed 2 weeks later. Mock-inoculations were made in the same way, but using sterile 2% MEA as inoculum. All inoculations were performed by the same person (RL) to increase uniformity.

### Data Collection

Seedling height was measured at the beginning and end of the experiment (to the nearest 0.5 cm) and seedlings were monitored for mortality at monthly intervals (four monitoring occasions throughout the experiment). Mortality was defined as discoloration of all needles above the inoculation point, consistent with previous studies (Krokene and Solheim, 1998; Jankowiak, 2006). At the conclusion of the experiment, the full length of the necrotic lesion on the outer bark (**Figure 1B**) was measured with an electronic caliper (to the nearest 0.01 mm), and the bark was peeled back to expose the sapwood (xylem). The full length of the necrotic lesion along the sapwood surface (**Figure 1C**) was measured in the same way. The stem was then cut in half at the upper edge of the inoculation site, and the stem diameter and lesion depth (**Figure 1D**) were measured.

To verify infections, re-isolations were made from 25 randomly selected inoculated seedlings from each treatment (in total 75 seedlings) at the conclusion of the experiment by cutting small sapwood samples from around the inoculation sites. These samples were plated on 2% MEA, incubated at 25°C, and inspected for fungal growth for up to 2 weeks.

### Statistical Analyses

General linear mixed models (GLMM) were constructed to evaluate the effects of the climate change scenarios on bark and sapwood lesion lengths and depth at the experiment conclusion. Due to inherent imprecision in measuring lesion length/depth in dead wood, seedlings that died throughout the experiment (53 seedlings) were excluded from the analyses. Significant outliers were removed (Grubb’s test, *P* < 0.05) and data were natural log transformed (lesion lengths) to approximate normality. Initial explanatory variables for the bark and sapwood lesion length models were seedling height at the onset of the experiment (continuous variable), climate change scenario, fungal inoculation strain (both categorical variables), and all interactions. Seedling tray number (categorical), position in the tray (three categories; rows/columns ranging from the outside to the interior) and the intercept were set as random factors. The same model was conducted for lesion depth, with seedling height replaced by stem diameter. Since the lesions of mock-inoculated seedlings very rarely extended inward from the inoculation point (only two very minor cases were identified), these data were removed from the analyses. All models were sequentially reduced, whereby non-significant interaction terms were removed if their removal did not increase AIC by > 2 units (Littell et al., 2006). Model comparisons were made using the maximum likelihood (ML) method and final values were obtained using restricted maximum likelihood (REML). Model assumptions were visually checked from the residual distribution.

To facilitate interpretation of a three-way interaction between seedling height at the onset of the experiment, fungal strain and climate change scenario in the sapwood lesion length model (*F*_climate scenario × fungal strain × beg_height 10,357_ = 1.81, *P* = 0.057), a mixed model was constructed with sapwood lesion length explained by height at the onset of the experiment. Tray number, position in the tray and the intercept were again set as random factors. The residuals of this model were then used as the response variable in a linear mixed model in which sapwood lesion length was explained by fungal strain, climate change scenario and their interaction, as per the methods described above.

Due to the low overall proportion of seedling deaths, the interactive effects of climate change scenario and fungal strain on mortality levels could not be evaluated using a linear model approach. Instead chi-squared tests were used for pair-wise comparisons of mortality counts between the three climate change scenarios (summing all fungal strains). Generalized linear mixed models, with a binary distribution and logit link function, were constructed to separately evaluate the effects of climate change scenario on mortality at the end of the experiment (dichotomous outcome; alive or dead) for seedlings inoculated with the two fungal strains that produced the greatest number of dead seedlings (F4 and F5). Again seedling tray number, position in the tray and the intercept were included as random factors. Chi-squared tests were used to compare within-strain mortality counts between climate change scenarios for the other fungal strains, excluding mock-inoculations, which had no mortality.

Pearson correlation tests were used to assess the relationship between outer bark and xylem sapwood lesion length, lesion depth and seedling mortality. For these comparisons, the estimated marginal means of bark and sapwood lesion length (from the seedling height adjusted model) and lesion depth for each climate change scenario/strain combination were compared against each other and the respective group seedling mortality counts. Statistical analyses were conducted using SAS version 9.3 (SAS Institute Inc., Cary, NC, United States).

## Results

**FIGURE 2:**
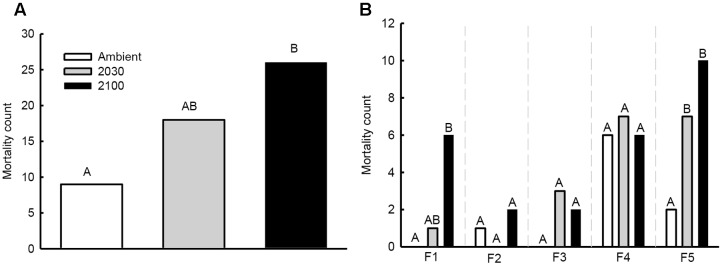
**Seedling mortality counts at the experiment conclusion:** **(A)** all fungal strains combined, and **(B)** differences between fungal strain as identified in generalized linear models (strains F4 and F5) and chi-squared tests (strains F1, F2, and F3). Different letters above counts represent statistically significant (*P* < 0.05) differences among climate change scenarios.

Total seedling mortality during the experiment was 12% (53/450), and ranged from 0% in the mock-inoculated group to 25% of seedlings inoculated with fungal strains F4 and F5 (**Figures 2A,B**). Ninety-two percentage of seedling mortality occurred during the initial 2 months of the experiment. More seedling deaths occurred under the 2100 climate change scenario than under ambient conditions (26 versus 9 deaths; χ^2^ value = 9.35, *P* = 0.002; **Figure 2A**). No significant difference was detected between the 2030 and 2100 climate change scenarios (χ^2^ value = 1.70, *P* = 0.192).

For seedlings inoculated with fungal strain F5, mortality varied based on the climate change scenario (**Table 1** and **Figure 2B**). More deaths occurred in seedlings assigned to the 2100 (pair-wise difference *P* = 0.011) and 2030 (*P* = 0.050) climate change scenarios than ambient conditions. No difference was detected between 2030 and 2100 climate change scenarios. Conversely, no variation in seedling mortality occurred under climate change scenarios using the F4 fungal strain (**Table 1** and **Figure 2B**). For strain F1, mortality was greater in the 2100 climate change scenario than ambient conditions (Fisher’s exact test, two-sided *P* = 0.022), but did not vary between 2030 and 2100 or between ambient conditions and the 2030 climate change scenario. No mortality differences were detected between climate change scenarios for seedlings inoculated with strains F2 or F3 (**Figure 2B**).

**Table 1:** Most parsimonious models to explain response variables (general and generalized linear mixed models).

Response	Source of variation	Num. d.f	Den. d.f	*F*	*P*
Mortality – strain F5	**Climate scenario**	**2**	**66**	**3.40**	**0.039**
Mortality – strain F4	Climate scenario	2	66	0.08	0.923
Bark lesion length	Onset height	1	358	0.00	0.946
	Climate scenario	2	374	1.74	0.177
	Fungi strain	5	374	44.29	<0.001
	**Scenario × strain**	**10**	**373**	**3.82**	**<0.001**
Sapwood lesion length	Climate scenario	2	375	8.36	<0.001
	Fungi strain	5	375	142.62	<0.001
	**Scenario × strain**	**10**	**375**	**4.82**	**<0.001**
Lesion depth	**Stem width**	**1**	**299**	**77.09**	**<0.001**
	Climate scenario	2	299	0.31	0.732
	Fungi strain	4	299	28.15	<0.001
	**Scenario × strain**	**8**	**299**	**3.36**	**0.001**


*Initial lesion models included all interactions between the sources of variations. Sapwood lesion length is adjusted by height at the experiment onset (see Materials and Methods). Highest order interactions or effects are bolded.*

**FIGURE 3:**
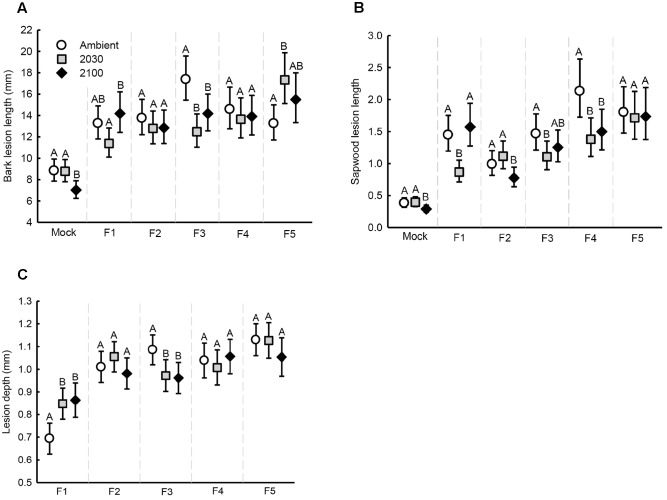
**Estimated marginal means (error bars depict 95% confidence intervals) of seedling lesions at the experiment conclusion in relation to climate scenarios and fungal strain:** **(A)** bark lesion length, **(B)** sapwood lesion length (adjusted by seedling height at the onset of the experiment and therefore an index relative to each other; see Materials and Methods), and **(C)** lesion depth. Different letters above error bars represent statistically significant (*P* < 0.05) within-strain differences.

Bark lesion length (**Table 1** and **Figure 3A**), sapwood lesion length (**Figure 3B**) and lesion depth (**Figure 3C**) all varied based on the interaction between climate change scenario and the fungal strain. A suite of within-strain differences in lesion length and depth due to climate change scenarios were identified, which are indicated in the figures. Lesion depth increased with seedling diameter (**Table 1**). Bark and sapwood lesion lengths were greater across all fungal strains than with mock-inoculations (statistical comparison was not possible for lesion depth, as lesions from mock-inoculations rarely extended into the sapwood). Mean sapwood lesion length (35.60 mm, range 4.9 – 139.4 mm) was longer than the mean bark lesion length (13.7 mm, range 4.5 – 36.0 mm).

**FIGURE 4:**
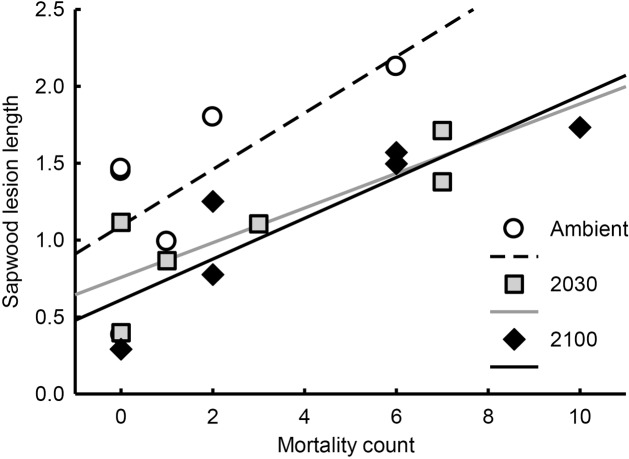
**Relationship between sapwood lesion length index (estimated marginal means) and seedling mortality counts (overall correlation *r* = 0.60, *P* = 0.009).** Each point represents the mortality count/mean sapwood lesion length for each strain/treatment group combination. Note that sapwood lesion length is an index adjusted by seedling height at the beginning of the experiment.

Both bark (*r* = 0.54, *P* = 0.022) and sapwood (*r* = 0.60, *P* = 0.009; **Figure 4**) lesion lengths were positively correlated with seedling mortality counts, while lesion depth was not. Bark and sapwood lesion length were not correlated with each other (Pearson correlation coefficient 0.92, *P* < 0.001). The depth and bark or sapwood lesion lengths were not correlated.

Minimal seedling growth occurred during the experimental period with no growth recorded for 243 of 450 seedlings (growth range 0 to 2 cm). *E. polonica* was successfully re-isolated from 74 of the 75 tested seedlings at the experiment conclusion.

## Discussion

Results of this study show that outcomes of fungal infections may vary between current environmental conditions and future climate change predictions in the Norway spruce – *E. polonica* pathosystem. While the most distant and severe projections were the most detrimental to tree health overall, this trend masked substantial between-strain variation in responses to the projected climate scenarios. This is the first experimental evaluation of climate change effects on this pathosystem, and indicates potential implications for future forestry productivity in northern Europe.

All of the fungal strains used in this study were pathogenic, and able to induce greater seedling damage than the mock-inoculations. Most fungal strains were from the same locality (Linnakoski et al., 2016), and the among-strains differences in response to climate change scenarios (e.g., the mortality differences between strains F4 and F5; **Figure 2B**), are thus consistent with previous observations that *E. polonica* populations can include strains of varying aggressiveness (Krokene and Solheim, 1998; Lieutier et al., 2003; Sallé et al., 2005). The bark beetle vector is able to disperse over long distances (Nilssen, 1984; Forsse and Solbreck, 1985), and thereby accumulate fungal assemblages from diverse geographical areas. However, this finding highlights problems associated with generalizing results across and within pathogen and host species, and thus the need for targeted system-based research.

Although potential new phenotypic and genetic markers have been developed, methods to accurately measure tree resistance/susceptibility and fungal pathogenicity remain challenging (Kovalchuk et al., 2013; Krokene et al., 2013). Presently, measurements of necrosis length are the most commonly used markers (Christiansen and Horntvedt, 1983; Horntvedt et al., 1983; Solheim, 1988; Krokene and Solheim, 1998; Kirisits and Offenthaler, 2002). In this study, the lesion length indices were effective general predictors for seedling mortality, with sapwood lesion length the most accurate. By also evaluating mortality, which was facilitated by the high seedling numbers, we uncovered substantial within-strain discordance between the lesion indices and mortality counts. For example, the mortality count was greater under the 2100 climate scenario than ambient conditions for seedlings infected with fungi F5, but none of the lesion indices differed between the two climate scenarios. These results thus indicate a need for caution when extrapolating lesion indices as more general outcomes of plant health. From a practical point of view, mortality occurred in 25% of seedlings inoculated with fungal strain F5 (compared to 0% without fungal infection). Twenty-five percentage of stand loss alone will have severe economic implications, and we found that mortality levels approached 50% under the 2100 climate scenario (**Figure 2B**).

Previous research showing the inability of Norway spruce to respond to environmental changes (Sallas et al., 2003; Kellomäki et al., 2008; Lévesque et al., 2013; Kroner and Way, 2016) suggests that seedling resistance (rather than fungal aggressiveness) primarily mediate the observed effects of climate scenarios on seedling health. However, this hypothesis calls for future targeted research addressing indices of plant immunity. Although conifers have highly evolved defense mechanisms against herbivores and pathogens (Krokene et al., 2013), several climate change factors are likely to contribute to plant stress and thereby impair their defenses (Julkunen-Tiitto et al., 2015). Previous studies have investigated the potential impact of elevated temperature and CO_2_ levels on the carbon based (phenolics and terpenoids) defense chemistry of Norway spruce (Sallas et al., 2003; Julkunen-Tiitto et al., 2015), indicating that climatic factors are likely to induce changes in the phenolic and monoterpene concentrations.

Norway spruce genotypes (clones) vary in their resistance to fungal infections (Swedjemark and Stenlid, 1997; Keriö et al., 2015; Lundén et al., 2015). Outbred seedlings were used for the present study, representing the natural genetic diversity that is currently employed in boreal forestry practices. Monocultures of resistant clones would provide a possible solution to combat specific infection risk. However, the subsequent lack of genetic diversity would also introduce other risks to tree health, specifically the ability to respond to unanticipated environmental perturbations such as droughts and other infections (Wingfield et al., 2015).

Forestry is a major economic industry in northern Europe. Much research has addressed the effects of projected climate changes on tree species distributions and their productivity (Kellomäki et al., 2008; Lévesque et al., 2013), while the potential impacts of pests and pathogens have received far less attention. Our results highlight the need to incorporate disease effects into future forestry planning. They suggest that predicted climate changes have the potential to alter the damage caused to Norway spruce by the fungus *E. polonica*, but that such effects can vary markedly among fungal strains. However, we acknowledge the multifaceted effects of climate change on plant-pathogen interactions. Factors such as volatile organic compounds (VOCs), gaseous emissions and radiation may have important influences (Niinemets et al., 2013), and we encourage further empirical research on this topic.

## Author Contributions

RL, MW, PP, and FA designed the experiment. RL collected the data. KF contributed to parts of the data collection and conducted the statistical analyses. MW, PP, and FA contributed reagents, materials and analysis tools. RL and KF drafted the manuscript; MW, PP, and FA were involved in the results interpretation and finalization of the manuscript. All authors listed have made substantial, direct and intellectual contribution to the work, and approved it for publication.

## Conflict of Interest Statement

The authors declare that the research was conducted in the absence of any commercial or financial relationships that could be construed as a potential conflict of interest.
