# Supplementary material for: Testing Projected Climate Change Conditions on the Endoconidiophora polonica / Norway spruce Pathosystem Shows Fungal Strain Specific Effects
Source: Front Plant Sci. 2017 May 26;8:883. doi: 10.3389/fpls.2017.00883 (PMC5445173; doi:10.3389/fpls.2017.00883)
Supplement: Supplementary file 1 [file Table_1.pdf]

Table S1. *Endoconidiophora polonica* strains used in this study.

Strain	CBS no[1]	Host tree	Vector	Origin	Date of collection	Collected by	Isolated by	Identified by
F1	142280	*Picea abies*	*Ips typographus*	Vodlajärvi, Russia	July 2005	H. Roininen	J. Ahtiainen	R. Linnakoski
F2	142307	*P. abies*	phoretic mite	Ruokolahti, Finland	26 October 2013	S. Mahilainen	S. Mahilainen	R. Linnakoski
F3	142281	*P. abies*	*I. typographus*	Ruokolahti, Finland	9 June 2013	S. Mahilainen	S. Mahilainen	R. Linnakoski
F4	142282	*P. abies*	*I. typographus*	Ruokolahti, Finland	5 June 2013	S. Mahilainen	S. Mahilainen	R. Linnakoski
F5	142283	*P. abies*	*I. typographus*	Ruokolahti, Finland	9 June 2013	S. Mahilainen	S. Mahilainen	R. Linnakoski

[1] Culture collection the Westerdijk Fungal Biodiversity Institute (CBS), Utrecht, The Netherlands
